# A large CRISPR-induced bystander mutation causes immune dysregulation

**DOI:** 10.1038/s42003-019-0321-x

**Published:** 2019-02-18

**Authors:** Dimitre R. Simeonov, Alexander J. Brandt, Alice Y. Chan, Jessica T. Cortez, Zhongmei Li, Jonathan M. Woo, Youjin Lee, Claudia M. B. Carvalho, Alyssa C. Indart, Theodore L. Roth, James Zou, Andrew P. May, James R. Lupski, Mark S. Anderson, F. William Buaas, Daniel S. Rokhsar, Alexander Marson

**Affiliations:** 1 Biomedical Sciences Graduate Program, University of California, San Francisco, CA 94143 USA; 2 Department of Microbiology and Immunology, University of California, San Francisco, CA 94143 USA; 3 Diabetes Center, University of California, San Francisco, CA 94143 USA; 4 Innovative Genomics Institute, University of California, Berkeley, CA 94720 USA; 5 Department of Chemistry, University of California, Berkeley, CA 94720 USA; 6 Department of Pediatrics, University of California, San Francisco, CA 94143 USA; 7 Department of Molecular and Human Genetics, Baylor College of Medicine, Houston, TX 77030 USA; 8 Department of Biomedical Data Science, Stanford University, Stanford, CA 94305 USA; 9 Chan Zuckerberg Biohub, San Francisco, CA 94158 USA; 10 Genetic Engineering Technologies, The Jackson Laboratory, Bar Harbor, ME 04609 USA; 11 Department of Molecular and Cell Biology, University of California, Berkeley, CA 94720 USA; 12 Okinawa Institute of Science and Technology, Okinawa, 904-0495 Japan; 13 Department of Medicine, University of California, San Francisco, CA 94143 USA; 14 UCSF Helen Diller Family Comprehensive Cancer Center, University of California, San Francisco, CA 94158 USA

## Abstract

A persistent concern with CRISPR-Cas9 gene editing has been the potential to generate mutations at off-target genomic sites. While CRISPR-engineering mice to delete a ~360 bp intronic enhancer, here we discovered a founder line that had marked immune dysregulation caused by a 24 kb tandem duplication of the sequence adjacent to the on-target deletion. Our results suggest unintended repair of on-target genomic cuts can cause pathogenic “bystander” mutations that escape detection by routine targeted genotyping assays.

## Abstract (web summary)

Dimitre Simeonov, Alexander Brandt et al. report a pathogenic bystander mutation caused by unintended repair of a CRISPR-Cas9-mediated deletion in mice. They generate mice lacking an *IL2RA* intronic enhancer previously associated with human disease risk and find that one line of edited mice show unexpected disease features due to a bystander mutation.

## Introduction

CRISPR-Cas9 genome engineering is employed widely to generate targeted in vitro and in vivo genetic modifications^1^. The Cas9 nuclease can be programmed to target specific genome sequences via a short guide RNA. Although unintended genome alterations have been mitigated by recent technical advances^2–6^, they remain a concern, especially for therapeutic applications of CRISPR. To date, attention has been focused on “off-target” editing in which Cas9 nuclease activity is directed towards genomic sites, other than the target, with varying degrees of homology to the guide RNA. Here we demonstrate that “bystander” mutations—unintended mutations neighboring the “on-target” cut site—must also be considered.

## Results

### CRISPR-Cas9 deletion of *Il2ra* enhancer

**Fig. 1:**
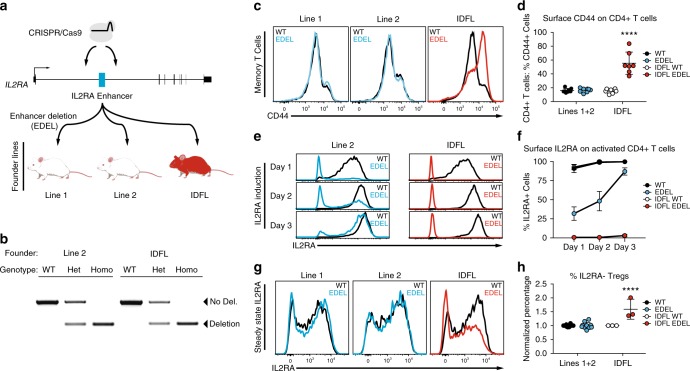
Immune dysregulation in a founder line of CRISPR-engineered *Il2ra* enhancer deletion mice. **a** CRISPR-engineered *Il2ra* enhancer deletion (EDEL) founder lines that were bred for immunophenotyping. **b** Genomic DNA PCR to genotype the *Il2ra* enhancer deletion in animals from Line 2 and the immune dysregulated founder line (IDFL). **c** Representative CD44 surface staining on CD4+ T cells isolated from spleens of wild-type (WT) and EDEL mice from different founder lines. **d** Quantification of percent CD44+ cells from (**c**) (Lines 1 and 2: WT *n* = 8, EDEL *n* = 7; IDFL: WT *n* = 8, EDEL *n* = 7). **e** Representative induction of IL2RA surface expression on naive CD4+ T cells (CD4+IL2RA-CD44–) activated with anti-CD3/CD28 antibodies. **f** Quantification of percent IL2RA+ cells from (**e**) (Line 2: WT *n* = 4, EDEL *n* = 4; IDFL: WT *n* = 4, EDEL *n* = 4). **g** Representative IL2RA surface expression on FOXP3+CD4+T cells (Tregs) from spleen of different founders. **h** Quantification of normalized percent IL2RA- cells of CD4+FOXP3+ Tregs from (g) (Lines 1 and 2: WT *n* = 10, EDEL *n* = 10; IDFL: WT *n* = 3, EDEL *n* = 3). Panels (**d**) and (**h**) include data from Lines 1 and 2 animals previously published^7^. All data are presented as mean ±s.d. and are representative of at least two independent experiments. ****P ≤ 0.001 by two-way analysis of variance (ANOVA) with Dunnett’s multiple comparisons test. Raw gel image corresponding to **b** is shown in Supplementary Figure 7

One advantage of genome editing over RNA knock-down approaches is that non-coding sequences can be modified, which enables studies of non-coding variants commonly associated with human disease risk. We recently identified a conserved autoimmunity-associated *IL2RA* intronic enhancer that controls the timing of gene expression in response to T-cell stimulation^7^. To study its in vivo function, we used CRISPR to engineer non-obese diabetic (NOD) mice with deletion of this enhancer (EDEL). We successfully generated EDEL founder lines by targeting Cas9 to cut on either side of the ~360-bp enhancer (Fig. 1a). Genomic PCR and targeted Sanger sequencing confirmed that approximately 360–370 bp was deleted at the enhancer site in multiple founders (Fig. 1b, Supplementary Figure 1). Three of the founders were backcrossed to wild-type NOD animals at least one generation before breeding the enhancer deletion to homozygosity for experimentation.

Surprisingly, immunophenotyping revealed a marked systemic difference in one line of mice. Unlike the other two characterized lines, homozygous EDEL progeny from the third founder line had hallmark features of a lymphoproliferative disorder, including variable splenomegaly, increased cellularity and higher percentages of memory T cells (Figs. 1c, d and Supplementary Figure 1). Despite the phenotypic differences among the lines, the on-target enhancer deletion only differed by a few nucleotides at the margins of the deletion (Supplementary Figure 1). The evolutionarily conserved DNA sequence at the site was deleted in all three lines and the line with more severe phenotype had a slightly smaller deletion, suggesting that the genotyped sequence differences directly at the deletion site did not explain observed differences in immune regulation (Supplementary Figure 1). The more severe immune phenotype persisted in progeny with the enhancer deletion from the affected line, even after an additional round of backcrossing and multiple generations of breeding, suggesting a mutation in close genomic proximity to the on-target deletion site rather than an unlinked off-target effect. Taken together, our data suggested the presence of an additional mutation linked to the *Il2ra* enhancer deletion in this immune dysregulated founder line (IDFL).

To determine the molecular and cellular effects of the linked mutation in the IDFL mice, we analyzed IL2RA expression. Double-negative (DN) thymocytes from IDFL mice had marked loss of IL2RA expression, whereas DN thymocytes from the other lines of EDEL mice had normal IL2RA expression (Supplementary Figure 1). Mature CD4+ effector T cells (Teffs) normally upregulate IL2RA to their surface after activation. Strikingly, in vitro stimulated IDFL Teffs largely failed to express IL2RA on their surface (Figs. 1e, f and Supplementary Figure 2). This was in contrast to the other EDEL lines, which showed delayed but not ablated induction of IL2RA following stimulation of naive T cells^7^. We also examined FOXP3+ regulatory T cells (Tregs), which constitutively express high levels of IL2RA and require it for their survival. Across lymphoid tissues there was an increased percentage of FOXP3+IL2RA– Tregs in IDFL mice compared with other EDEL lines (Figs. 1g, h and Supplementary Figure 2). In vitro and in vivo regulatory T-cell differentiation were impaired (Supplementary Figure 3). Interestingly, a subset of T cells, including some Tregs, did express high levels of IL2RA. An *Il2ra* null mutation would be expected to ablate expression across cell types. Instead, we find that the linked mutation has effects on IL2RA expression that vary among cells, with a subset of T cells selectively maintaining IL2RA expression.

### Identification of a bystander mutation

**Fig. 2:**
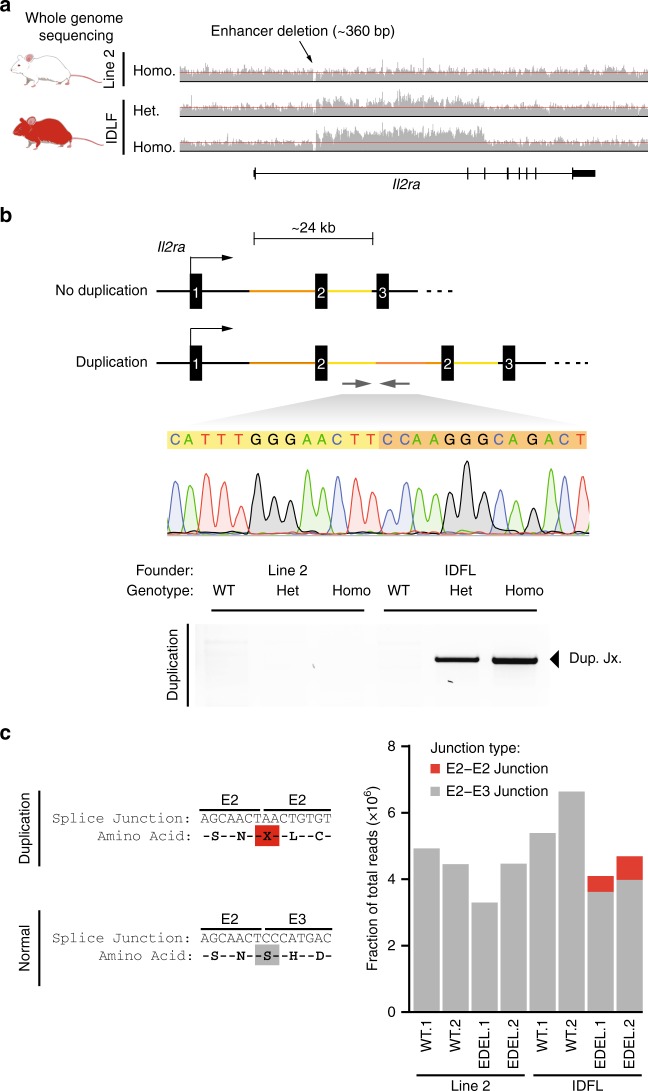
Identifying a large tandem duplication in the *Il2ra* locus. **a** Read pileups at the *Il2ra* locus from genome sequencing of a homozygous enhancer deletion (EDEL) mouse (Line 2) and homozygous and heterozygous EDEL mice from the immune dysregulated founder line (IDFL). Red lines were added to highlight the elevated read counts in IDFL mice. **b** Schematic of the *Il2ra* locus with the large tandem duplication in IDFL mice. PCR and Sanger sequencing across the novel junction sequence created by the duplication. **c** Read counts from RNA sequencing of IL2RA+CD4+T cells showing reads that span the aberrant exon 2–exon 2 junction in red and the normal exon 2–exon 3 junction in gray. Data in (**c**) are from biological replicates derived from two independent experiments. Raw gel image corresponding to **b** is shown in Supplementary Figure 7

To identify the mutation causing marked immune dysregulation, we sequenced the whole genomes of EDEL mice from the IDFL and from one of the other founder lines (Fig. 2a). We looked for a causative IDFL mutation both at the *IL2ra* locus and throughout the genome. Consistent with the observed genetic linkage with the enhancer deletion, we discovered a large structural mutation in the *Il2ra* locus that was unique to the IDFL genome. Careful analysis of the read pileups revealed a 24-kb block of DNA with elevated coverage in the IDFL genome compared with adjacent sequences, consistent with an increase in copy number (Fig. 2a). Paired-end reads at the breakpoint implied a tandem duplication, which we confirmed by genomic PCR and Sanger sequencing (Fig. 2b). The duplicated sequence starts immediately downstream of the deleted *Il2ra* enhancer and spans the remainder of the first intron, the second *Il2ra* exon and most of the second intron. This unexpected structural mutation tightly linked to the intended on-target edit is a “bystander” mutation that causes marked immune dysregulation.

We next interrogated how the duplication formed. Previous work showed that paired CRISPR-induced DNA breaks can result in tandem duplication of the intervening sequence^8,9^, however, no predicted off-target cutting sequences were identified in the *Il2ra* locus to explain this duplication event (Supplementary Figure 4). Although we cannot rule out spontaneous DNA breaks from DNA replication, our data suggest that the duplication is more likely an unintended product of repair from on-target editing. Sequence homology could contribute to a duplication event. We did not find extended sequence homology between the cut site and the duplication junction, but we did discover microhomology at the breakpoint junction (Supplementary Figure 4). However, three nucleotides of microhomology are found commonly in the genome, raising a question of why this distal site may have been used for repair. Chromatin looping can bring distal genomic sites into close proximity. Indeed, published high-resolution chromatin conformation capture data suggested three-dimensional proximity between the *Il2ra* enhancer and the site of the duplication junction (Supplementary Figure 4)^10^, although the statistical significance of this putative looping interaction was not determined. We then tested whether microhomology and looping were sufficient to drive recurrent duplications at this site (Supplementary Figure 4). We repeated CRISPR microinjections into single-cell NOD/ShiLtJ zygotes, cultured them in vitro and analyzed > 50 blastocysts and failed to observe the recurrence of this particular duplication event despite efficient deletion (~80%) of the enhancer at the on-target site. Note, there were two blastocysts with PCR bands of roughly the expected duplication size, but the sequence could not be confirmed. Taken together, our analysis of the duplication is consistent with a complex unintended repair consequence that occurs much less frequently than the intended enhancer deletion.

The duplication of exon 2 is predicted to generate a novel splice junction that would result in a premature stop codon in the *Il2ra* mRNA (Fig. 2c). Although the expected effect of the homozygous duplication would be to ablate protein expression in every cell, we were nevertheless able to detect CD4+ T cells with near normal levels of IL2RA expression. To understand how these cells expressed IL2RA despite the predicted premature stop codon generated by the duplication, we performed RNA-seq on IL2RA+ T cells from spleen. As expected, we could identify reads with the aberrant exon 2–exon 2 splice junction only in the IFDL EDEL cells (Fig. 2c and Supplementary Figure 5). However, IL2RA+ IDFL cells had ~10-fold more reads that contained the wild-type exon 2–exon 3 junction than the aberrant exon 2–exon 2 junction (Fig. 2c). PCR and Sanger sequencing of *Il2ra* complementary DNA (cDNA) confirmed that these cells predominantly generate *Il2ra* transcript with a single exon 2 between exons 1 and 3 (Supplementary Figure 5). In contrast, we did not detect transcripts with a single exon 2 in IL2RA– T cells induced to express *Il2ra* (Supplementary Figure 5). These isoform differences suggest that some T cells, including a subset of Foxp3+ Tregs, are able to correctly splice the aberrant *Il2ra* genetic structure and productively translate IL2RA.

## Discussion

This report links a CRISPR-induced bystander mutation to in vivo pathology. We build on previous work that showed CRISPR editing can cause bystander deletions and complex rearrangements in neighboring on-target sequences^11^. Further work is needed to understand the frequency with which such mutations occur, as well as the DNA repair rules that underlie these events. Identification of the bystander mutation depended on having multiple independent founder pedigrees to demonstrate an aberrant phenotype in one line and genetically link it to the on-target edit. New methods and analytical tools are needed to detect both unintended CRISPR-induced bystander and off-target mutations. Genome engineering not only allows gene knockout, but also permits targeted alterations of non-coding *cis*-regulatory sequences for mechanistic study of human variants and for cell therapies. The bystander mutation allele observed here was introduced by murine zygote editing by Cas9 mRNA and gRNA microinjection. The marked immune phenotype was revealed by breeding the rare allele to homozygosity. The functional consequences, if any, of rare unintended alleles in a population of human primary somatic cells edited by various CRISPR delivery strategies remain largely untested. Bystander editing effects—which can be easily missed with conventional genotyping methods—must be carefully assessed for research and clinical CRISPR applications, especially for mounting therapeutic efforts to fine tune gene regulatory programs.

## Methods

### Mouse generation

*Il2ra* enhancer deletion (EDEL) mice were generated by the Jackson Laboratory (Bar Harbor, ME, USA) by microinjection of gRNA and Cas9 mRNA. Briefly, Cas9 mRNA (100 ng µl^−1^) and mIL-2Ra-CaRE4 gRNAs (50 ng µl^−1^) were mixed and injected into NOD/ShiLtJ zygotes. Four founders with the enhancer deletion were identified by PCR amplicon size and confirmed by sequencing of TOPO-cloned PCR products. We immunophenotyped three founder lines. The EDEL mouse lines were established by backcrossing founders for at least one generation before breeding to homozygosity. Protospacer sequence for gRNAs used in the production of the founder lines in this study are in Supplementary Data 1.

### Mouse genotyping

The enhancer deletion was initially genotyped in all founders by Sanger sequencing genomic DNA from proteinase K digested tail tissue. PCR amplification of the CaRE4 enhancer was carried out using HotStart Taq (Bioline USA Inc.) and primers (mIl2ra-EDEL-F, mIl2ra-EDEL-R) that span the edited site. PCR amplicons were then sequenced with the mIl2ra-EDEL-F primer. Primer sequences in Supplementary Data 1. The duplication junction was confirmed by PCR amplification of the junction followed by gel electrophoresis and Sanger sequencing. The primers used are in Supplementary Data 1.

### Mouse experiments and data analysis

All mice were maintained in the UCSF-specific pathogen-free animal facility in accordance with guidelines established by the Institutional Animal Care and Use Committee and Laboratory Animal Resource Center. Experiments were done with animals aged between 1 and 4 months. Wild-type littermate mice were used as controls for all immunophenotyping experiments. All mice used in this study were normoglycemic. No data were excluded from analysis. Power calculations were not performed and data were assumed to be normally distributed. Experiments were done without blinding or randomization. All data are derived from at least two independent experiments unless otherwise stated. Both male and female mice were used for experiments.

### Mouse immunophenotyping

Briefly, cells from spleen, peripheral lymph nodes (peri-LNs), and thymus were collected from each mouse. Spleen, peri-LNs, and thymus were dissociated in 1× phosphate-buffered saline (PBS) with 2% fetal bovine serum (FBS) and 1 mM EDTA. The mixture was then passed through a 70-μm filter. Ammonium-chloride-potassium lysis buffer was used to deplete red blood cells from splenocytes. All antibody stains were performed at a 1:100 dilution in 30–50 μl of 1× PBS. To pellet the cells, centrifugation was performed at 300 *g* for 5 min. For immunophenotyping, approximately 2 million cells were stained per tissue sample. Cells were first stained with a viability dye at a 1:1000 dilution in 1× PBS for 20 min at 4 °C, then washed with EasySep Buffer (1× PBS, 2% FBS, 1 mM EDTA). Cells were then resuspended in the appropriate surface staining antibody cocktail and incubated for 30 min at 4 °C, then washed with 1× PBS. Cells were then fixed, permeabilized, and stained for transcription factors using the Foxp3 staining kit (eBioscience) according to the manufacturer’s instructions. Antibody staining panels are listed in Supplementary Data 1.

### IL2RA induction on stimulated naive T cells

Naive T cells were isolated from spleen and lymph nodes with CD4+ negative selection (Stemcell Technologies) followed by fluorescence-activated cell sorting for CD4+IL-2Ra−CD44−. In all, 100,000 cells were activated per well of a 96-well plate coated with 2 μg ml^−1^ anti-CD3 and anti-CD28. Cell analysis by flow cytometry was performed every day for 3 days.

### In vitro Treg differentiation

Spleens were dissociated in EasySep Buffer and splenocytes were enriched for CD4+ T cells using the Mouse CD4 Negative Selection Kit as described above. Naive CD4 + CD62L + CD44– T cells were sorted. In all, 100,000 cells were stimulated with plate bound anti-CD3/CD28 antibodies in a 96-well plate in the presence of 2 ng/ml TGF-b (Miltenyi Biotec) and 200 U/ml IL2 (Miltenyi Biotec) for 3 days. Treg differentiation was assessed by surface staining for IL2RA and intracellular staining for Foxp3 without a viability dye.

### Genome sequencing sample preparation

DNA was isolated from kidney tissue by phenol–chloroform extraction. PCR-free whole-genome libraries were constructed by the Genome Technologies Core at The Jackson Laboratory using the KAPA Hyper Prep Kit (KAPA Biosystems), targeting an insert size of 400 base pairs. Libraries were checked for quality and concentration using the Bioanalyzer High Sensitivity DNA Assay (Agilent), Qubit dsDNA BR Assay (ThermoFisher), and quantitative PCR (KAPA Biosystems), according to the manufacturer's instructions. Libraries were sequenced at Novogene, 150-bp paired-end on the HiSeq X (Illumina) to a target mean coverage depth of 30 × .

### Genome sequencing alignment and variant calls

Bwa mem was used for alignment to the mouse mm10 reference sequence. Reads at the identified tandem duplication junctions were then assembled using Velvet^12^ in order to confirm the exact base pair sequence, as well as assist in picking primers for confirmation of the duplication via PCR. The Picard Software Suite and GATK 4.0 pipeline^13^ with default settings was used for variant analysis. Base recalibration was performed with NOD-specific variants, both single nucleotide polymorphisms (SNPs) and insertions and deletions (INDELs), obtained from the Wellcome Sanger Institute Mouse Genome Project.

### CRISPR off-target analysis

We first performed a biased off-target analysis looking for variants 5 bp on either side of predicted off-target cut sites. This was done for the top 49 predicted off-target sites for each gRNA that was used to make the enhancer deletion mouse lines. In total, six variants were found with this analysis, all of which were present in the NOD background variant panel. We also performed an unbiased variant analysis to examine potentially confounding mutations that fit a likely inheritance model. The cohort variant call file was subset for biallelic SNPs and INDELs where all individuals were assigned a genotype with a sufficient average coverage ( > = 10 reads). Variants were unique or in excess in the immune dysregulated mouse as compared with the other two mice in the cohort. The resulting alleles were further subset by removing NOD-specific SNP and INDEL variants and selecting only variants that fell within exonic regions. This revealed 2407 variants. The remaining variants are likely specific to the NOD mice used to generate our founder lines. Other than the 24-kb duplication, we found no coding or splicing mutations near the enhancer deletion that might contribute to the observed phenotypes. Identifiers GT_05102 and GT_05105 refer to heterozygous and homozygous EDEL mice from the IDFL founder line. GT05111 is homozygous EDEL mouse from Line 2. Finally, we performed computational prediction of off-target cutting for the *Il2ra* enhancer gRNAs throughout the *Il2ra* gene body (Supplementary Figure 4).

### RNA sequencing

Briefly, approximately 500,000 CD4 + IL2RA + cells were sorted from CD4-enriched splenocytes and total RNA was isolated from samples using the RNeasy Micro Kit (QIAGEN) according to the manufacturer’s instructions with the following options: cells were pelleted and resuspended in RLT buffer with β-mercaptoethanol and homogenized using QIAshredder (QIAGEN). DNA removal was performed with gDNA Eliminator Columns (QIAGEN). RNA samples were analyzed with a NanoDrop spectrophotometer and all samples had a 260/280 ratio of 1.80 or higher. RNA integrity was measured by Bioanalyzer and all samples had an RNA integrity score (RIN) of 8.0 or more. RNA-seq libraries were prepared by the Functional Genomics Laboratory at Berkeley. RNA samples were poly-A selected and then converted into sequencing libraries with the ultra-low input SMART-seq kit. The samples were pooled and sequenced on one lane of the Illumina HiSeq4000. *Il2ra* isoform analysis was done using the UNIX grep command to identify reads in raw fastq that contained sequences for the E2–E2 junction (ACCAGCAACTAACTGTGTCT) or E2–E3 junction (ACCAGCAACTCCCATGACAA). Read counts were normalized to the total number of reads for a given sample. Short reads were also aligned with STAR to the mouse mm10 reference. Differential expression analysis was performed using EdgeR from Bioconductor Package for R^14^.

### *Il2ra* cDNA isoform analysis

In all, 500,000 IL2RA+ (CD4+IL2RA+) and IL2RA– (CD4+IL2RA+CD44–) CD4+ T cells were sorted from CD4-enriched splenocytes. IL2RA– CD4+ T cells were stimulated in vitro for 10 h with 2 μg ml^–1^ plate-bound anti-CD3/CD28 antibodies (Biolegend). In total, 430,000 DN thymocytes (Live CD45+CD4–CD8–) were sorted from thymus. RNA was extracted using the RNA Micro Kit (QIAGEN) as described above. RNA was reverse transcribed into cDNA using SuperScript VILO MasterMix as per manufacturer’s protocol (Thermo Scientific). PCR of the cDNA to assess exon 2 splicing was performed with forward and reverse primers that sit in *Il2ra* exon 1 and exon 3, respectively (Supplementary Data 1). PCR was carried out with Bioline Taq 2x MasterMix as per manufacturer’s protocol. Amplicons were cut out of the agarose gel and purified using QIAGEN’s Gel Extraction Kit. Amplicons were sequenced in the forward and reverse directions using the primers from the initial PCR amplification.

### Zygote *Il2ra* enhancer editing

NOD/ShiLtJ zygotes were microinjected with gRNAs and Cas9 mRNA, matching the generation of *Il2ra* EDEL mice. PCR amplification and Sanger sequencing were used to check gDNA for the duplication junction and WT *Il2ra* sequence upstream of *Il2ra* exon 3, the genomic site of the IDFL duplication junction (Supplementary Figure 4). Blastocyst gDNA was also checked for on-target enhancer deletion by PCR amplification and Sanger sequencing (Supplementary Figure 4). A second test for the duplication junction was performed using a nested PCR. Briefly, duplication junction PCR samples were diluted (1 μl sample in 10 μl water) and 1 μl of dilution was used as the template. A nonspecific band was observed in a majority of the blastocysts (data not shown). Nine amplicons were extracted and sent for Sanger sequencing, of which six were successfully sequenced. The primers used for these assays are listed in Supplementary Data 1. The reconstructed sequences are assembled in Supplementary Figure 6. Takara PrimeSTAR polymerase was used for amplification with 30-s extension for 35 cycles. PCR products were run on agarose gel for size separation and visualization.

### Data analysis

The statistical tests and sample sizes used for data analysis are included in figure legends.

### Illustrations

Images in Fig. 1a (mice) and Supplementary Figure 4 (scissors) were created with BioRender.

### Reporting summary

Further information on experimental design is available in the Nature Research Reporting Summary linked to this article.

## Supplementary information

Descriptions of Additional Supplementary Files

Reporting Summary

Supplementary Data 1

Supplementary Data 2

Supplementary Information

## Data Availability

Whole-genome sequencing and RNA sequencing data have been uploaded to NCBI Sequencing Read Archive (SRA Accession: PRJNA510427). These data were used to generate Figs. 2a,  c. Additional raw data used to generate the main figures are available in Supplementary Data 2. Any other relevant data in this manuscript will be made available upon request.

## References

[1] Sander JD, Joung JK (2014). CRISPR-Cas systems for editing, regulating and targeting genomes. Nat. Biotechnol..

[2] Kleinstiver BP (2016). High-fidelity CRISPR–Cas9 nucleases with no detectable genome-wide off-target effects. Nature.

[3] Doench JG (2016). Optimized sgRNA design to maximize activity and minimize off-target effects of CRISPR-Cas9. Nat. Biotechnol..

[4] Slaymaker IM (2016). Rationally engineered Cas9 nucleases with improved specificity. Science.

[5] Guilinger JP, Thompson DB, Liu DR (2014). Fusion of catalytically inactive Cas9 to FokI nuclease improves the specificity of genome modification. Nat. Biotechnol..

[6] Cong L (2013). Multiplex genome engineering using CRISPR/Cas systems. Science.

[7] Simeonov DR (2017). Discovery of stimulation-responsive immune enhancers with CRISPR activation. Nature.

[8] Boroviak K, Fu B, Yang F, Doe B, Bradley A (2017). Revealing hidden complexities of genomic rearrangements generated with Cas9. Sci. Rep..

[9] Li J (2015). Efficient inversions and duplications of mammalian regulatory DNA elements and gene clusters by CRISPR/Cas9. J. Mol. Cell Biol..

[10] Bonev B (2017). Multiscale 3D genome rewiring during mouse neural development. Cell.

[11] Kosicki, M., Tomberg, K. & Bradley, A. Repair of double-strand breaks induced by CRISPR-Cas9 leads to large deletions and complex rearrangements. *Nat. Biotechnol*. https://doi.org/10.1038/nbt.4192 (2018). 10.1038/nbt.4192 PMC6390938 30010673

[12] Zerbino DR, Birney E (2008). Velvet: algorithms for de novo short read assembly using de Bruijn graphs. Genome Res..

[13] McKenna A (2010). The Genome Analysis Toolkit: a MapReduce framework for analyzing next-generation DNA sequencing data. Genome Res..

[14] Robinson MD, McCarthy DJ, Smyth G (2010). K. edgeR: a Bioconductor package for differential expression analysis of digital gene expression data. Bioinformatics.

