# Supplementary material for: A large CRISPR-induced bystander mutation causes immune dysregulation
Source: Commun Biol. 2019 Feb 18;2:70. doi: 10.1038/s42003-019-0321-x (PMC6379443; doi:10.1038/s42003-019-0321-x)
Supplement: Supplementary file 1 — Descriptions of Additional Supplementary Files [file 42003_2019_321_MOESM1_ESM.docx]

**Description of Additional Supplementary Files**

**File Name**: Supplementary Data 1

**Description**: Reagents used in this study.

**File Name**: Supplementary Data 2

**Description**: Raw data used to generate main figures.
