# Supplementary material for: A large CRISPR-induced bystander mutation causes immune dysregulation
Source: Commun Biol. 2019 Feb 18;2:70. doi: 10.1038/s42003-019-0321-x (PMC6379443; doi:10.1038/s42003-019-0321-x)
Supplement: Supplementary file 2 — Reporting Summary [file 42003_2019_321_MOESM2_ESM.pdf]

natureresearch

Corresponding author(s): *Double-blind peer review submissions: write DBPR and your manuscript number here instead of author names.*

Reporting Summary

Nature Research wishes to improve the reproducibility of the work that we publish. This form provides structure for consistency and transparency in reporting. For further information on Nature Research policies, see Authors & Referees and the Editorial Policy Checklist.

Statistical parameters

When statistical analyses are reported, confirm that the following items are present in the relevant location (e.g. figure legend, table legend, main text, or Methods section).

n/a	Confirmed	
☐	☒	The exact sample size (*n*) for each experimental group/condition, given as a discrete number and unit of measurement
☒	☐	An indication of whether measurements were taken from distinct samples or whether the same sample was measured repeatedly
☒	☐	The statistical test(s) used AND whether they are one- or two-sided<br>*Only common tests should be described solely by name; describe more complex techniques in the Methods section.*
☒	☐	A description of all covariates tested
☐	☒	A description of any assumptions or corrections, such as tests of normality and adjustment for multiple comparisons
☐	☒	A full description of the statistics including central tendency (e.g. means) or other basic estimates (e.g. regression coefficient) AND variation (e.g. standard deviation) or associated estimates of uncertainty (e.g. confidence intervals)
☐	☒	For null hypothesis testing, the test statistic (e.g. *F*, *t*, *r*) with confidence intervals, effect sizes, degrees of freedom and *P* value noted<br>*Give P values as exact values whenever suitable.*
☒	☐	For Bayesian analysis, information on the choice of priors and Markov chain Monte Carlo settings
☒	☐	For hierarchical and complex designs, identification of the appropriate level for tests and full reporting of outcomes
☒	☐	Estimates of effect sizes (e.g. Cohen's *d*, Pearson's *r*), indicating how they were calculated
☐	☒	Clearly defined error bars<br>*State explicitly what error bars represent (e.g. SD, SE, CI)*

Our web collection on statistics for biologists may be useful.

Software and code

Policy information about availability of computer code

Data collection	HiSeq Control Software
Data analysis	Burrows-Wheeler Aligner (BWA) short read aligner, STAR RNA-seq aligner, Genome Analysis Tool Kit (GATK), PINDEL structural variant analyzer

For manuscripts utilizing custom algorithms or software that are central to the research but not yet described in published literature, software must be made available to editors/reviewers upon request. We strongly encourage code deposition in a community repository (e.g. GitHub). See the Nature Research guidelines for submitting code & software for further information.

Data

Policy information about availability of data

All manuscripts must include a data availability statement. This statement should provide the following information, where applicable:

- Accession codes, unique identifiers, or web links for publicly available datasets
- A list of figures that have associated raw data
- A description of any restrictions on data availability

Whole genome sequencing and RNA sequencing data will be uploaded to NCBI GEO upon acceptance of this manuscript.

Field-specific reporting

Please select the best fit for your research. If you are not sure, read the appropriate sections before making your selection.

☒ Life sciences ☐ Behavioural & social sciences

For a reference copy of the document with all sections, see nature.com/authors/policies/ReportingSummary-flat.pdf

Life sciences

Study design

All studies must disclose on these points even when the disclosure is negative.

Sample size	Sample sizes of at least 3 or more animals were used for mouse experiments unless otherwise noted. Genome sequencing data is based on individual mice. RNA-sequencing performed on 2 biological replicates per genotype per founder line.
Data exclusions	No data were excluded from the study.
Replication	All conclusions made from mouse data are based on at least two independent experiments with a minimum of three animals, unless otherwise stated.
Randomization	No randomization was performed in this study.
Blinding	Experimentors were not blinded to the genotypes of the mice used in this study.

Materials & experimental systems

Policy information about availability of materials

n/a	Involved in the study
☒	☐ Unique materials
☐	☒ Antibodies
☒	☐ Eukaryotic cell lines
☐	☒ Research animals
☒	☐ Human research participants

Antibodies

Antibodies used	A full list of antibodies and related information is included in Supplementary Table 1.
Validation	All antibodies used in this study have been previously described and used in previously published research.

Research animals

Policy information about studies involving animals; ARRIVE guidelines recommended for reporting animal research

Animals/animal-derived materials	Mice in this study were generated at Jackson Laboratories on the NOD ShiltJ background (Stock#001976). Male and female mice between 1-4 months of age were used for experiments.

Method-specific reporting

n/a	Involved in the study
☒	☐ ChIP-seq
☐	☒ Flow cytometry
☒	☐ Magnetic resonance imaging

Flow Cytometry

Plots

Confirm that:

☒ The axis labels state the marker and fluorochrome used (e.g. CD4-FITC).

☒ The axis scales are clearly visible. Include numbers along axes only for bottom left plot of group (a 'group' is an analysis of identical markers).

☒ All plots are contour plots with outliers or pseudocolor plots.

☒ A numerical value for number of cells or percentage (with statistics) is provided.

Methodology

Sample preparation	All antibody stains were performed at a 1:100 dilution in 30 µl of 1x PBS. To pellet the cells, centrifugation was performed at 300g for 5 min. For immunophenotyping, approximately 2,000,000 cells were stained per tissue sample. Cells were first stained with a viability dye at a 1:1,000 dilution in 1x PBS for 20 min at 4 °C, then washed with EasySep Buffer (1x PBS, 2% FBS, 1 mM EDTA). Cells were then resuspended in the appropriate surface staining antibody cocktail and incubated for 30 min at 4 °C, then washed with 1x PBS. Cells were then fixed, permeabilized, and stained for transcription factors using the Foxp3 staining kit (eBioscience) according to the manufacturer's instructions. Antibody staining panels are listed in Supplementary Table 2.
Instrument	FACS Aria Fusion, Aria II, Fortessa and LSR II
Software	FACSDiva for collection and FlowJo (version 10) for analysis
Cell population abundance	500,000 regulatory and naive CD4+ T cells were sorted for RNA-seq. Approximately 1 million naive CD4+ T cells were sorted for Il2ra activation experiments. Post-sort purity was not directly assessed, however, characteristic proteins (Il2ra and CD69) were expressed by sorted cells following activation.
Gating strategy	Relevant gating strategies are included in Supplementary Information.

☒ Tick this box to confirm that a figure exemplifying the gating strategy is provided in the Supplementary Information.
