# Supplementary material for: A large CRISPR-induced bystander mutation causes immune dysregulation
Source: Commun Biol. 2019 Feb 18;2:70. doi: 10.1038/s42003-019-0321-x (PMC6379443; doi:10.1038/s42003-019-0321-x)
Supplement: Supplementary file 5 — Supplementary Information [file 42003_2019_321_MOESM5_ESM.pdf]

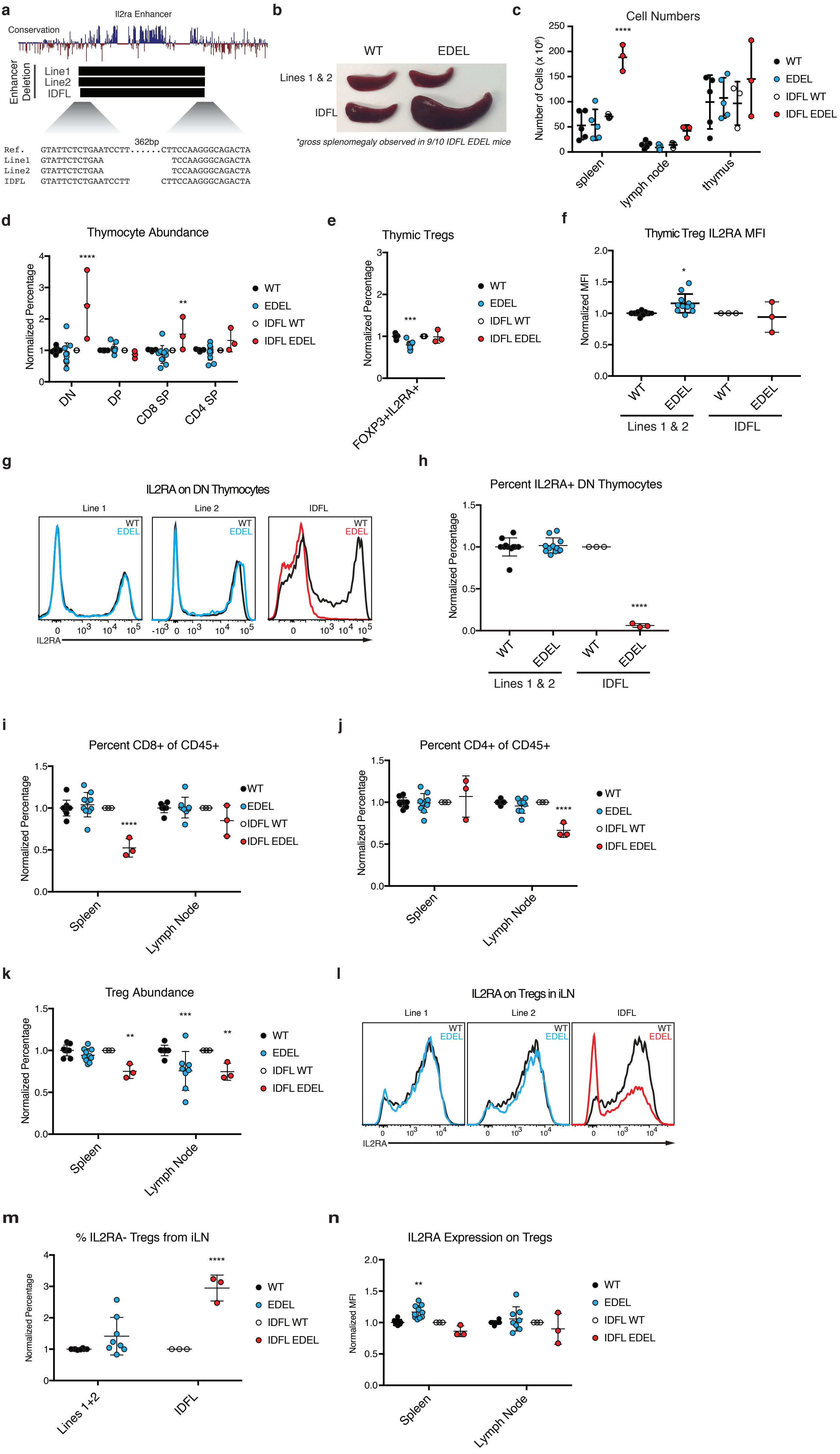

Supplementary Figure 1. Characterization of Enhancer Deletion Founder Lines. **a**, Genetic deletion of the conserved *Il2ra* enhancer showing the nucleotide sequence at the deletion breakpoints. **b**, Representative spleens from WT and EDEL mice derived from different founder lines. **c**, Cell counts from spleen, inguinal lymph nodes and thymus. **d**, Normalized percentage of double negative (DN), double positive (DP), CD8 SP and CD4 SP thymocytes of live CD45+ thymocytes (Lines 1 and 2: WT n=11, EDEL n=11; IDFL: WT n=3, EDEL n=3). **e**, Normalized percentage of FOXP3+IL2RA+ mature regulatory T cells (Tregs) of CD4 SP thymocytes (Lines 1 and 2: WT n=11, EDEL n=11; IDFL: WT n=3, EDEL n=3). **f**, Normalized IL2RA MFI on mature FOXP3+IL2RA+ Tregs (Lines 1 and 2: WT n=11, EDEL n=11; IDFL: WT n=3, EDEL n=3). **g**, Representative IL2RA surface expression on DN thymocytes from different founders. **h**, Normalized percentage of IL2RA+ DN cells of live CD45 + thymocytes. i, Normalized CD8+ percentage of live CD45+ cells in spleen and inguinal lymph nodes. **j**, Normalized CD4+ percentage of live CD45+ cells in peripheral lymphoid organs. **k**, Normalized percentage of FOXP3+ Tregs of CD4+ T cells in peripheral lymphoid organs. **l**, Representative IL2RA surface expression of FOXP3+ Tregs. **m**, Normalized percentage of IL2RA- FOXP3+ Tregs in peripheral lymphoid organs. **n**, Normalized IL2RA MFI on FOXP3+IL2RA+ Tregs in peripheral lymphoid organs. Data for Lines 1 and 2 includes animals for which immunophenotyping was previously published (Simeonov et al. Nature. 2017). Data in (c) derived from Lines 1 and 2: WT n=2, EDEL n=2 and IDFL: WT n=3, EDEL n=3. Data in (d,e,f,h) derived from Lines 1 and 2: WT n=11, EDEL n=11 and IDFL: WT n=3, EDEL n=3. Data in (m,n) derived from Lines 1 and 2: WT n=10, EDEL n=8 and IDFL: WT n=3, EDEL n=3. All data are presented as mean +/- s.d. and are representative of at least two independent experiments. *P ≤ 0.05, **P ≤ 0.01, ***P ≤ 0.001, ****P ≤ 0.001 by two-way ANOVA with Dunnett's multiple comparisons test.

a

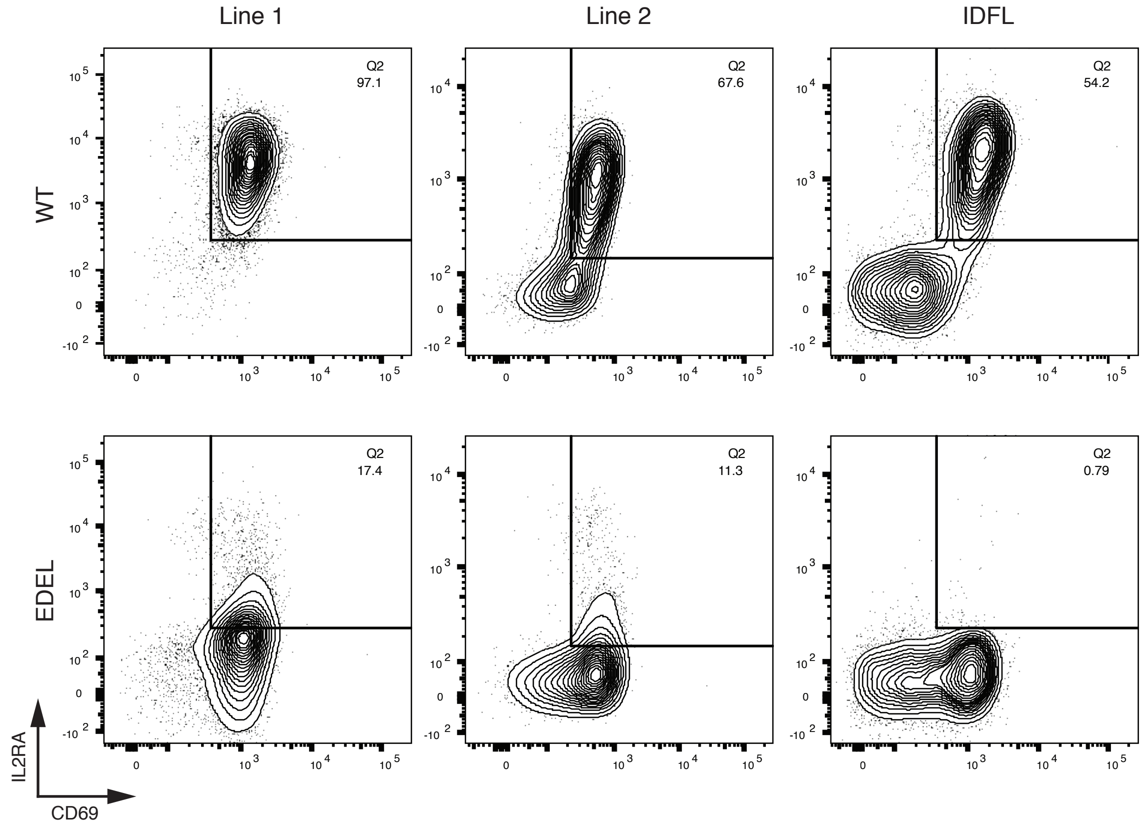

Supplementary Figure 2. IL2RA Surface Expression on Activated Naïve CD4+ T cells. a, IL2RA and CD69 surface expression on naïve CD4+ T cells (CD4+IL2RA-CD62L+CD44-) 24 hours after stimulation with anti-CD3/CD28 plate bound antibodies shown.

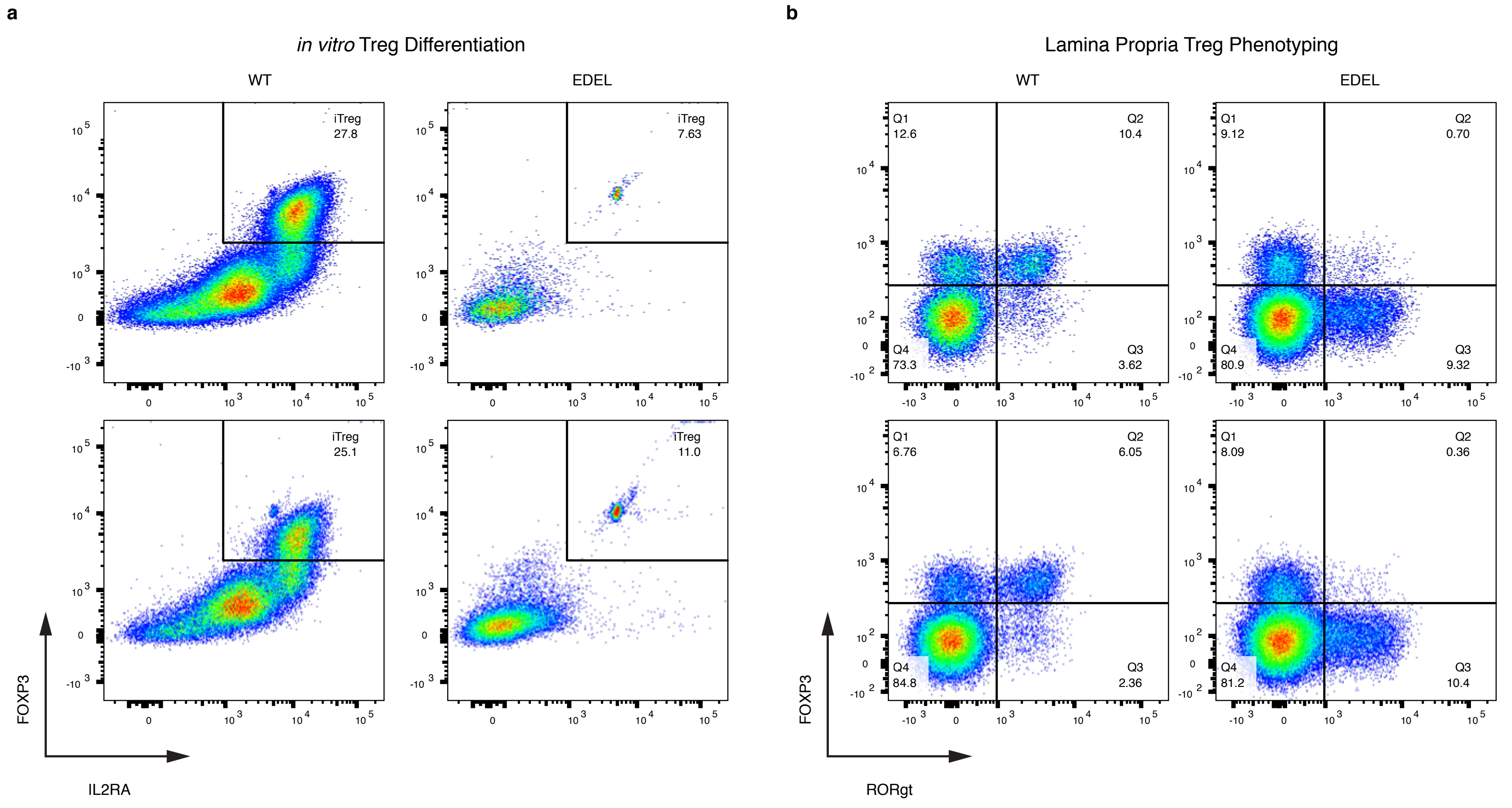

Supplementary Figure 3. Regulatory T cell characterization in vitro and in vivo. a, Expression of FOXP3 and IL2RA in IDFL naïve CD4+ T cells (CD4+IL2RA-CD62L+CD44-) in vitro differentiated to become regulatory T cell (Treg) for 3 days. **b**, FOXP3 and RORgt staining of IDFL CD4+ T cells (live CD45+TCRb+CD4+) from large intestine lamina propria. Data shown is from independent biological replicates and is derived from a single experiment.

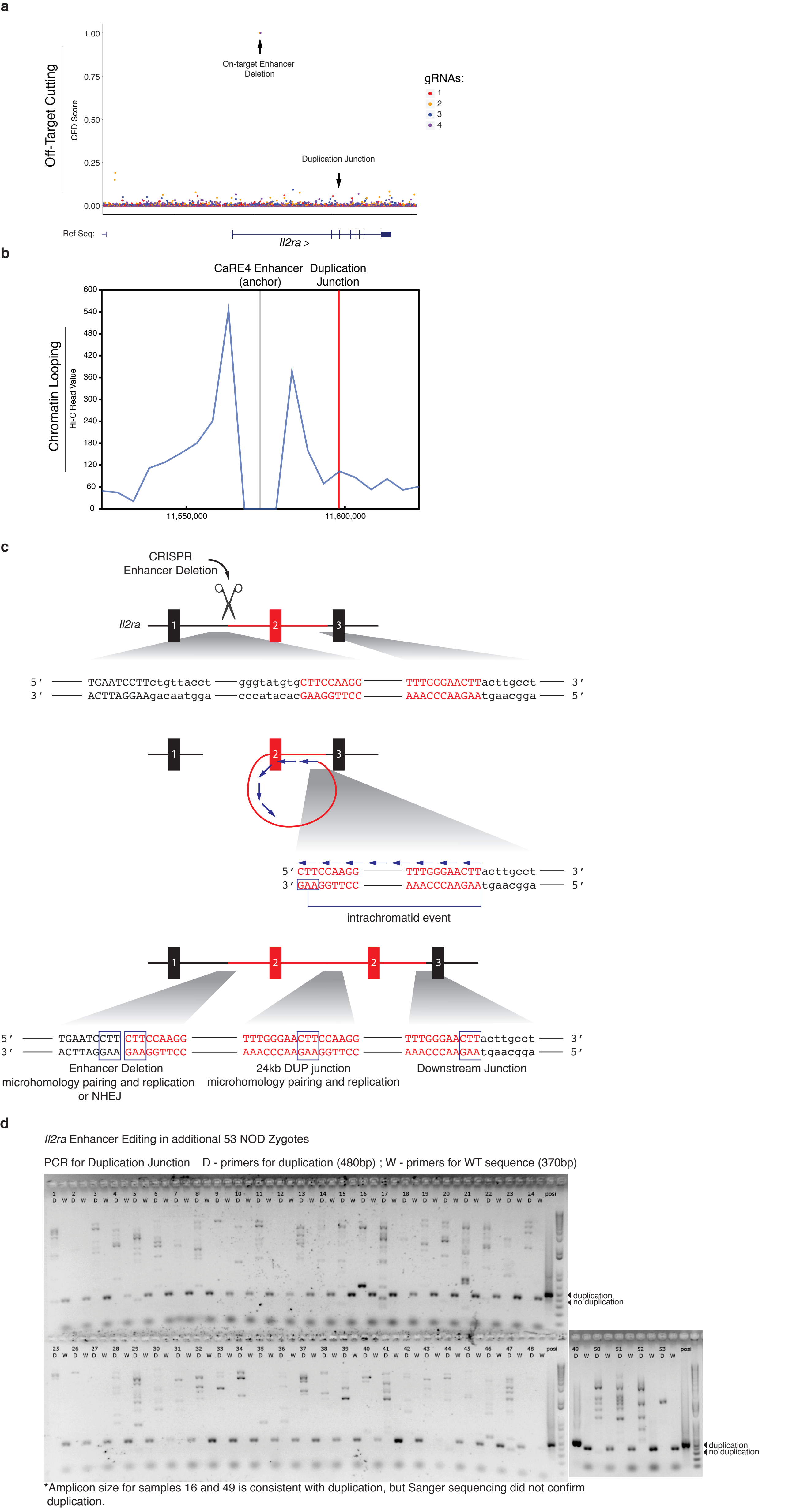

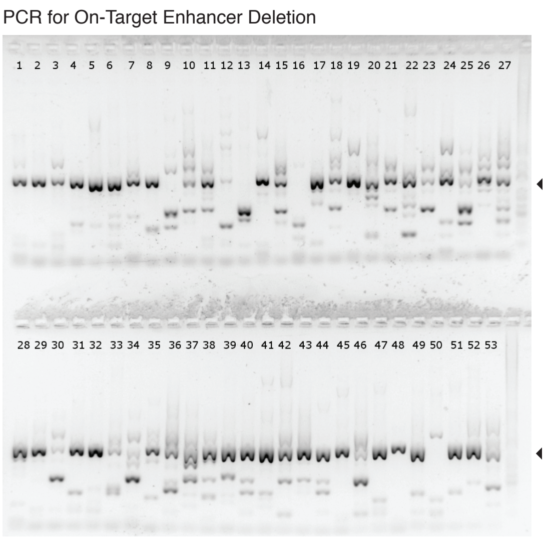

e

Samples	Sequencing Result/Homology
Blastocyst 5	non-specific, chr4, *Il2ra* enhancer
Blastocyst 6	sequencing failed
Blastocyst 10	non-specific, chr4
Blastocyst 11	non-specific, chr4
Blastocyst 22	sequencing failed
Blastocyst 42	sequencing failed
Blastocyst 44	chr2, no *Il2ra* sequences
Blastocyst 45	non-specific, chr2
Blastocyst 48	non-specific, chr1
IDFL gDNA (Ctrl)	*Il2ra* exon3, *Il2ra* enhancer

Supplementary Figure 4. Characterization of *Il2ra* bystander duplication. a, Computational prediction of off-target sites for the *Il2ra* enhancer gRNAs throughout the *Il2ra* gene body assessed by Continuous Frequency Determination (CFD) scoring (Doench and Fusi et al. 2016. Nat. Biotech.). We highlight the position of the duplication breakpoint. **b**, Overlap of the *Il2ra* locus with 5kb-resolution Hi-C data from mouse embryonic stem cells anchored at the *Il2ra* enhancer (Bonev et al. 2017. Cell). **c**, Schematic of proposed microhomology-mediated repair at the *Il2ra* locus that could generate the observed duplication (created with BioRender). **d**, PCR genotyping for *Il2ra* enhancer editing experiment in additional 53 microinjected NOD zygotes. Top, PCR results for the duplication breakpoint. Amplification was carried out for the duplication junction (D) or WT sequence (W). Bottom, PCR results for the on-target enhancer deletion. **e**, Summary of sequencing results from nested PCR to genotype IDFL duplication junction in enhancer-targeted NOD blastocysts. Fully reconstructed sequences available in Supplementary Information 1.

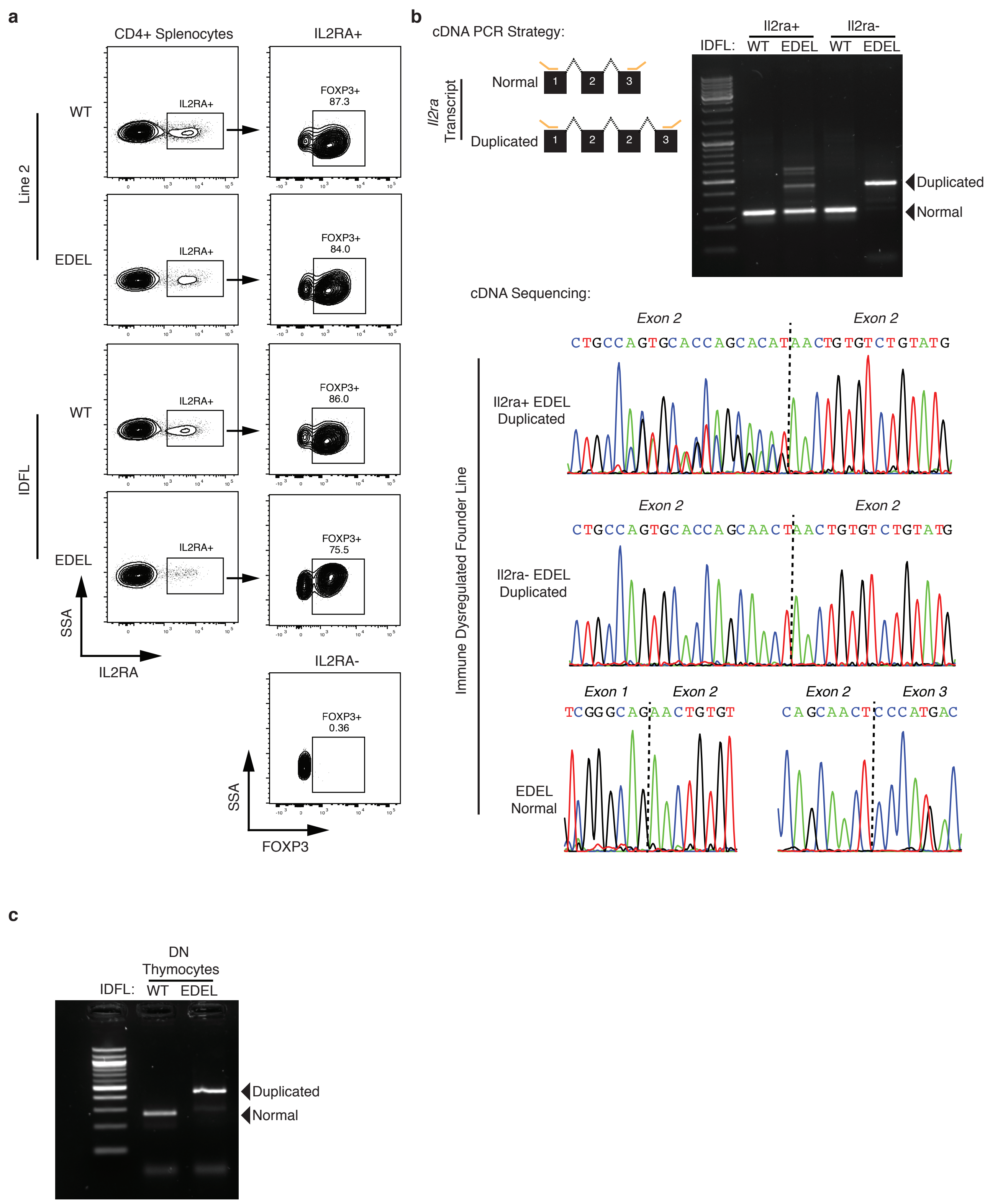

Supplementary Figure 5. *Il2ra* splicing analysis. a, Gating scheme used to sort IL2RA+ and IL2RA- cells. Foxp3 staining in sorted IL2RA+ cells. **b**, *Il2ra* exon 2 splicing analysis on IL2RA+ and stimulated IL2RA-CD44- cells in WT and EDEL CD4+ T cells from the immune dysregulated founder line (IDFL). PCR amplicons across exon 2 were generated from cDNA and run on a 1% agarose gel. The lower ("normal") band is consistent a single exon 2 *Il2ra* isoform, whereas the higher ("duplicated") band is consistent with an Il2ra isoform with two exon 2s. The amplicons were isolated and Sanger sequenced for verification. We observed that IL2RA+ IDFL EDEL cells showed variable larger amplicons of unknown significance. Sequencing these larger amplicons showed *Il2ra* transcript with two exon 2s, but did not explain the variable sizes observed. In these cells, we noted mixed Sanger sequencing peaks adjacent to the exon2-exon2 junction potentially consistent with an aberrant splice isoform or isoforms for which we do not know the significance. **c**, *Il2ra* exon 2 splicing analysis of IDFL WT and EDEL double negative (DN) thymocytes. The amplicons were isolated and Sanger sequenced for verification. We observed variable smaller amplicons of unknown significance. Sequencing these amplicons showed Il2ra transcript with normal splicing (WT) or two exon 2s (EDEL), but did not explain the variable sizes observed.

Primers for Assay

Il2raNstdF   5′ GCCATTTCTCATGCCTGTCT 3′
Il2raNstdR   5′ CTCAGCCCTTAGCTTGGGTA 3′

Positive Control

>IDFL gDNA (chr2:11Mb, *Il2ra* x3 and *Il2ra* enhancer; forward and reverse)
GCCATTTCTCATGCCTGTCTGGTAACAAGGTCCCTCATGCCCTACACAGGAGCGGTCTTTTGCTTATCTTTGCACACCCAGCACAGATATGTCTCTG
GGCACCAGAGAGGTGTCCCAGGGTAAATGACCGCATAGACAGCTCTTTCTATTCTGAACATTTGGGAACTTCCAAGGGCAGACTAAGAAGCCACTTA
CAGGTGAGTGTGACAGGTGAGAGGAACCGTGGCAAGGAACACCCTACCCTGGTGGCAGAGCTTCTCGATATTTGTATGTCTGCTGCTTTGCTTCTCA
GTTCATTCATGATCTATTGAGCAACTTCACGCAAGGTTAGAGAGGCAGAGATGAATAAGACCCAACTCTTGGCCTCCAGGAGCTTCTTGCTTATGCG
GAGAGAATACCCAAGCTAAGGGCTGAG

CRISPR-Targeted Blastocysts

>Blastocyst 5 (chr4:42Mb and chr2:11Mb; forward and reverse seq.)
TTCAAATGAAGATGTAGAACCCTCAGAGCTCCTTCTGAACCATGCCTGTCTGGATGCTGCCATGTTCTTGCCTTGGTGATAATGGATGACCTCTGAA
CCTGTAAGCCAGCCCCAGGTTAAATGTTGTCTTTTATAAGACTTGCATTGGTCATGGTGTCTGTTCACAGCAGTAAAACCCTAACTAAGACAGCTGC
TGAGCATGTTAATAGAAGACGGGTCTGTGTATCCCCCAAGCCAAGTAGAGTCTCCGTTTGCTAAACTATTTCCTATGCTTCCCTACCTCAC**TGTGTG**
TGTGTGTGTGTGTGTGTGTGTTCTCTCTCTCTCTCTCTCTCTCTCTCTCTCTCTCTCTCTCTTGGTAATAAAAAACAAAAGACAAAGATGGGTAT
GTGCAAGGGCAGACTAAGAAGCCACTTACAGGTGAGTGTGACAGGTGAGAGGAACCGTGGCAAGGAACACCCTACCCTGGTGGCAGAGCTTCTCGAT
ATTTGTATGTCTGCTGCTTTGCTTCTCAGTTCATTCATGATCTATTGAGCAACTTCACGCAAGGTTAGAGAGGCAGAGATGAATAAGACCCAACTCT
TGGCCTCCAGGAGCTTCTTGCTTATGCGGAGAGAATACCCAAGCTAAGGGCTGAG

>Blastocyst 10 (chr4:42M; forward only seq.)
TTCAAATGAAGATGTAGAACCCTCAGAGCTCCTTCTGAACCATGCCTGTCTGGATGCTGCCATGTTCTTGCCTTGCTTGGTGATAATGGATGAACCT
CTGAACCTGTAAGCCAGCCCCAGTTAAATGTTGTCTTTTATAAGACTTGCATTGGTCATGGTGTCTGTTCACAGCAGTAAAACCCTAACTAAGACAG
CTGCTGAGCATGTTAATAGAAGACGGGTCTGTGTATCCCCCAAGCTAAGGGCTGAG

>Blastocyst 11 (chr4:42M; forward only seq.)
AGAACCCTCAGAGCTCCTTCTGAACCATGCCTGTCTGGATGCTGCCATGTTCTTGCCTTGGTGATAATGGATGAACCTCTGACCTGTAAGCCAGCCC
CAGGTTAAATGTTGTCTTTTATAAGACTTGCATTGGTCATGGTGTCTGTTCACAGCAGTAAAACCCTAACTAAGACAGCTGCTGAGCATGTTAATAG
AAGACGGGTCTGTGTATCCCCCAAGCTAAGGGCTGAG

>Blastocyst 44 (chr2:31M; reverse only seq.)
AGGGGAGGGGTGTCCCAGAGAGCACACACCATGTAGTGGCATGGAGCCAAGTTTGTCCCTGCTTGGGTACAGGTGTCCCAGGTGTCCCAGCACGTAG
CTCTGGCCCAGGATGTCTGGCAGGGCAACTTCCCCTTGCCTTGAGGTGCCTTCCTGAATGTAGTGTGGGTGTGTGTGGGTGTCCAGGCATGGGAAAG
CTGGAGAAAAAGCCCTGTACTCATGTGCCCGCCCCTGTACCTCCGACAGCTTGGCCCCTCCTTACCCGGGGCTGGAGCCCTGCCGGTAGGTGGTAGG
ACAGGGCGTGTCTACACACTGGAAGCTGCCTCGGGTGTTGAAGCACATCTGGCCAGGCCCACACTCGATGCCATCTTCCTCACACTCGTTGATGTCT
GGGCGGTAGGGTGCGGTAGGAGATGGGAAGATAGAACAGGGGAGAGGGAGACCAGAGGAGGCCGTGTGAAAGAGACCAGCT

>Blastocyst 45 (chr2:130M, forward and reverse seq.)
ATTTGCCATTGTTTTCCAGGTTGGCTTGCTGGCCTGTGAGCCTGAGGTATCTGCCTGTCTTCCCCAGGGTTGGGGGTTACAGGCGTGTTCCAGTACA
CTCAGCCTTTGTGTGGAAGTTAGGTGTCTGAACTCAGATGTTCGAGATTATACATCAAATACTTGGCGCACCAACCCCTCTCCCAAGCTCAGACACA
CTTTCTTCTGGGCCTGTGCCAAGGTGAGGAAGCAGATGGGTGAGGACCAGGGCAGTAAGGAGGAAGCAGCTTCCAGGAACACGGGTGCAGCCTCTTG
TCTGCCACACACAGGTGGGAAATACATCGTGTCACTAGCTCCCACCCTGCTGAGCCCTGTCTATGTGCTGGCACGACTCTTCAGTCCTCTATCTTCC
CTATCTCTGAAATTTTTAGCTGCATACATCTTAAGACAAATTACCCAAGAGTTGAAAATAAGACCAAGAACAGAGGGGCTGTATCGTCTTAGGGGCT
GTGGAAGT

>Blastocyst 48 (chr1:64M; forward and reverse seq.)
AGGCAGACCTATTGCTCTTGGCTCGTTCTGCTCATGCCTCTCTGGGTCAGCTTTGCTCTAGGCTGGGTGGGGCTTCTGCAGTTTCCTTGGGGAAGCT
TTGCCTTCACACATCTCTCATCCCTTCCTGGAACCTTGGTTTCTTCTGGACATGACACATTCATCTCATGGAAAGAACAGAGATAGATACACGGAAG
AAAGCCAATCATAGCATGTTTTCTAGCCTGTACTTGTAGCATGTCTGTTAAAATCCTACTGGCCAAAGCAGGTGTTGCCATGAGAGCTAATGGGTGA
GGAGTATTCTCGACTCTTGTTGAAAGTGTGACACCTTCTATGGGCTTACTGACATAAAGAGGAATGAAAAATGCAGGTACTGGTGCACTCTGCTGCA
GCCTCCAGGCAGCAAGCCTTCTTGGTATCAGCTACTTTTTGTGCCCATCCCTTGAGATACAGGCCTTTCAAAGCCTGGTGTTCTCTCTGTTTAGGGT
TCTAAGCATGCAGGACAAACCAGACCAGGCAGAGAAGTGGCTACCCTCGCCAGTGACTTCAGCACAAAGGCTACACTTTGGCCTGCCTCCTAGGGTA
AACAGAGAAGGCTGGCAAGCCAGCACTGGCTCACGCCTCCTGGCTCTGGCTCCCTCCCCAGCTACCCCCACCCTAACAGCTTCTTAGAGTGTTAGCC
ACTGCTCTAGTGAGAGAAGAGTAATTGAGGGTGTAATTAAAAAAAAAAGAAGAAGAAGAAGGAGGAGG**AGGAGAAGGAGAAGGAGAAGGAGAAGGAG**
AAGGAGAAGGAGAAGGAGAAGGAGAAGGAGAAGGAGAAGGAGAAGGAGAAGGAGAAGGAGAAGGAGAAGGAGAAGGAGAAGAAGAAGAAGACATTAG
AGAGCAATAGACCAGATACCCCAGCTCAGGGCAGCTCTAAACCAAAAGACTAAGGCAG

Supplementary Figure 6. Nested PCR of enhancer-targeted blastocysts to detect IDFL duplication junction. Reconstructed amplicon sequences using Sanger sequencing and blast results in mouse genome (mm10). Nested primers for the IDFL duplication junction were used to genotype blastocysts. Underlined sequences show putative primer binding sites. Colored sequences represent genomic regions identified in Sanger sequencing. Orange - sequence adjacent to *Il2ra* exon 3; red - sequence adjacent to *Il2ra* enhancer; Additional colors – other genomic sequences.

a

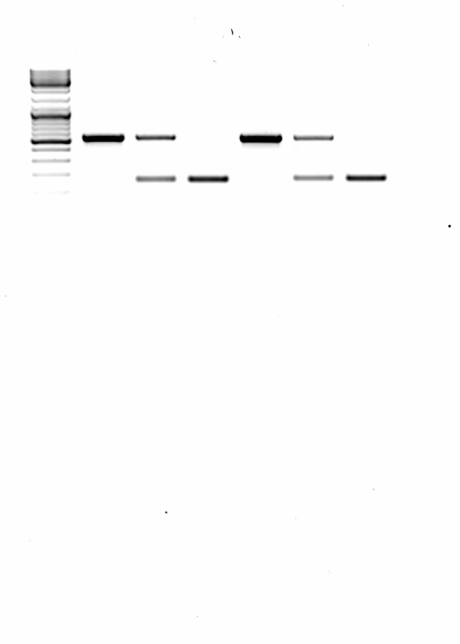

b

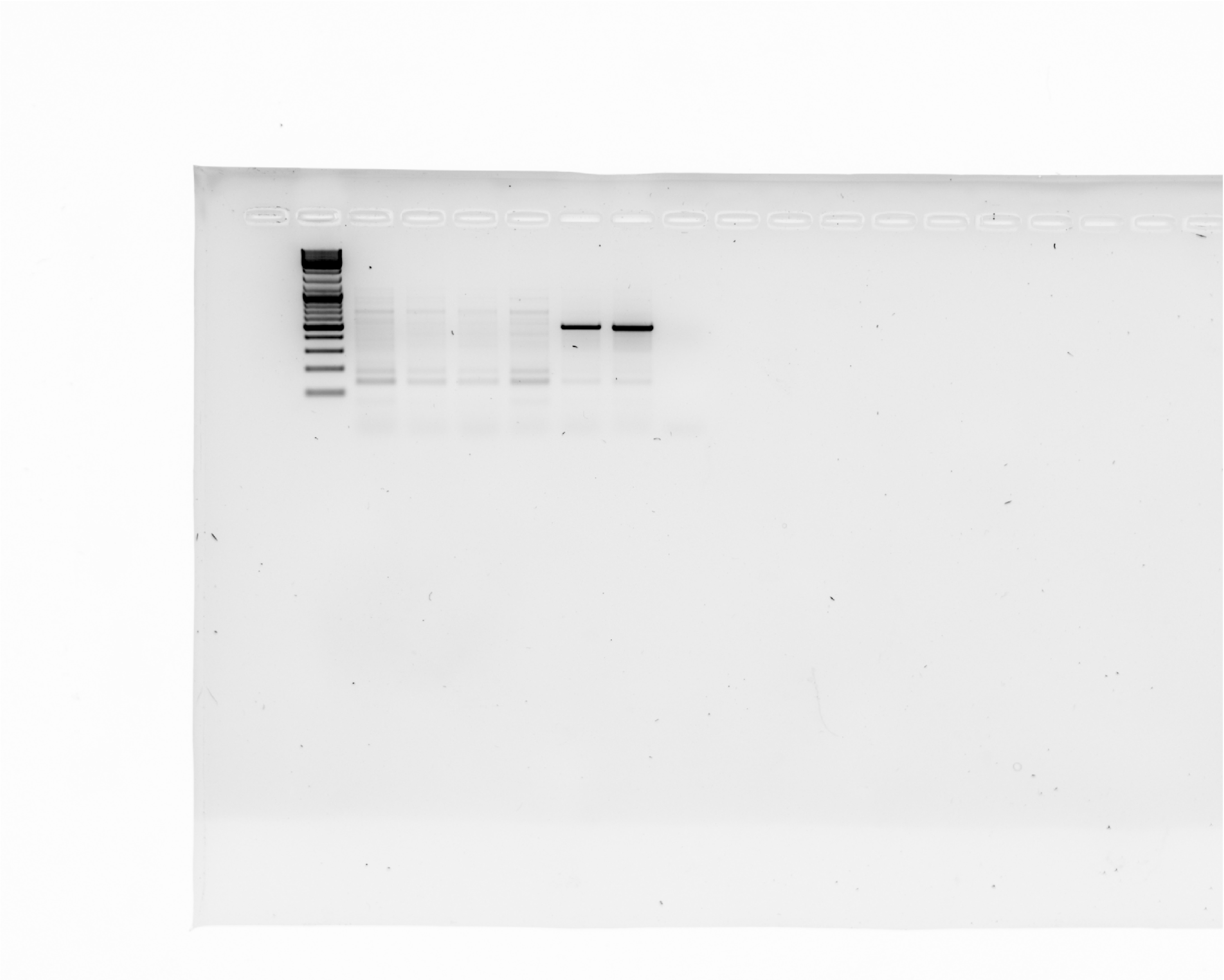

Supplementary Figure 7. a, Raw gel image for data in Figure 1b. **b**, Raw gel image for data in Figure 2b.
